# Born to sense: biophysical analyses of the oxygen sensing prolyl hydroxylase from the simplest animal *Trichoplax adhaerens*

**DOI:** 10.2147/HP.S174655

**Published:** 2018-11-09

**Authors:** Kerstin Lippl, Anna Boleininger, Michael A McDonough, Martine I Abboud, Hanna Tarhonskaya, Rasheduzzaman Chowdhury, Christoph Loenarz, Christopher J Schofield

**Affiliations:** Chemistry Research Laboratory, University of Oxford, Oxford, UK, christopher.schofield@chem.ox.ac.uk

**Keywords:** hypoxia, hypoxic response, oxygen sensing, hypoxia-inducible factor (HIF), evolution, dioxygenase, enzyme structure, PHD/EGLN prolyl hydroxylases, 2-oxoglutarate oxygenase, *Trichoplax adhaerens*

## Abstract

**Background:**

In humans and other animals, the chronic hypoxic response is mediated by hypoxia inducible transcription factors (HIFs) which regulate the expression of genes that counteract the effects of limiting oxygen. Prolyl hydroxylases (PHDs) act as hypoxia sensors for the HIF system in organisms ranging from humans to the simplest animal *Trichoplax adhaerens*.

**Methods:**

We report structural and biochemical studies on the *T. adhaerens* HIF prolyl hydroxylase (*Ta*PHD) that inform about the evolution of hypoxia sensing in animals.

**Results:**

High resolution crystal structures (≤1.3 Å) of *Ta*PHD, with and without its HIFα substrate, reveal remarkable conservation of key active site elements between *T. adhaerens* and human PHDs, which also manifest in kinetic comparisons.

**Conclusion:**

Conserved structural features of *Ta*PHD and human PHDs include those apparently enabling the slow binding/reaction of oxygen with the active site Fe(II), the formation of a stable 2-oxoglutarate complex, and a stereoelectronically promoted change in conformation of the hydroxylated proline-residue. Comparison of substrate selectivity between the human PHDs and *Ta*PHD provides insights into the selectivity determinants of HIF binding by the PHDs, and into the evolution of the multiple HIFs and PHDs present in higher animals.

## Introduction

**Figure 1:**
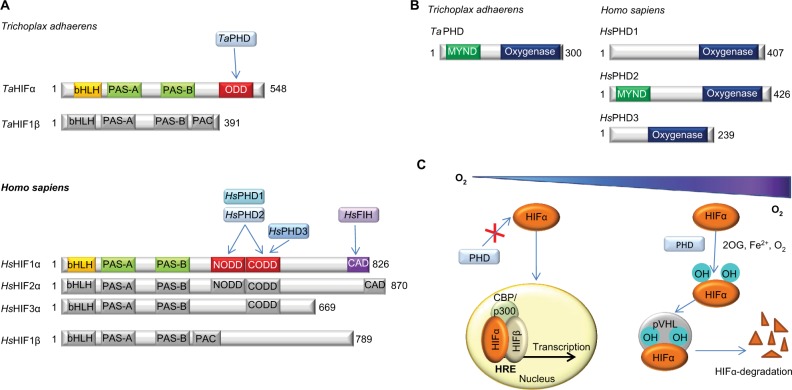
Overview of the HIF system. **Notes:** (**A**) Comparison of domain architectures of HIFα and HIFβ in *T. adhaerens* and humans. In contrast to the multiple PHD/HIFα-isoforms and ODDs present in vertebrates, the prime components of the HIF system in *T. adhaerens* involve only one PHD and one HIFα, which has a single ODD.^16^ Arrows indicate assigned prolyl and asparaginyl hydroxylation sites. (**B**) Domain structures of HIF prolyl hydroxylases (PHDs) in *T. adhaerens* and humans. Domain acronyms: 2OG dioxygenase domain (oxygenase), MYeloid, Nervy, and DEAF-1 (MYND)-type zinc finger domain (MYND). (**C**) Outline of the conserved mechanism of the response to chronic hypoxia in animals. The HIF transcription factors are regulated by PHD catalyzed hydroxylation of prolyl residues in the ODD(s) of HIFα under normoxia. Recognition of the hydroxylated prolyl residues by pVHL is followed by ubiquitination by the E3 ubiquitin ligase, which tags HIFα for proteasomal degradation. In humans, FIH, which is only sporadically present in non-vertebrate animals,^16^ constitutes an additional oxygen dependent regulatory element of HIF activity. FIH catalyzes the hydroxylation of an asparagine residue in the CTAD of HIFα, thus disrupting its interaction with the CBP/p300 transcriptional co-activator, and hindering transcriptional activation.^11^ **Abbreviations:** PHD, Prolyl hydroxylases; HIF, hypoxia-inducible transcription factor; ODD, oxygen-dependent degradation domain; bHLH, basic helix-loop-helix motif; PAS, Per-ARNT-Sim domain; CTAD, *C*-terminal transactivation domain; PAC, PAS-associated *C*-terminal domain; FIH, factor inhibiting HIF.

Hypoxia inducible transcription factors (HIFs) play key roles in maintaining oxygen homeostasis in most, if not all, animals.^1^–^4^ Extensive work on the HIF mediated response to hypoxia in humans and other higher animals (Figure 1A and B) has revealed that the levels and transcriptional activity of the α,β-heterodimeric HIF complex are regulated by ferrous iron and 2-oxoglutarate (2OG) dependent oxygenases.^4^–^6^ Prolyl-4-hydroxylation of the oxygen dependent degradation domains (ODDs) in HIFα is catalyzed by the HIF prolyl hydroxylases (PHDs or EGLNs), and signals for HIFα degradation via the ubiquitin proteasome system. HIFα prolyl hydroxylation strongly promotes its binding to the von-Hippel Lindau protein (pVHL), a targeting component of an E3 ubiquitin ligase complex^7^–^9^ (Figure 1C). In some animals, a second 2OG oxygenase, factor inhibiting HIF (FIH), catalyzes asparaginyl-hydroxylation in the *C*-terminal transcriptional activation domain (CTAD) present in both HIF1α and HIF2α, but not in HIF3α;^10^,^11^ this modification inhibits the interaction between HIF and transcriptional coactivators, which are histone acetyl transferases (CBP/p300).^11^ FIH is active at lower oxygen concentrations than the PHDs^12^,^13^ and is likely less important from a fundamental hypoxia-sensing perspective.

The PHDs and FIH belong to structurally distinct 2OG oxygenase subfamilies, with FIH being part of the Jumonji-C (JmjC) 2OG oxygenase subfamily IX,^14^ and the PHDs belonging to the PH (prolyl hydroxylase) subfamily VIII.^15^ However, unlike the HIFα-PHD-pVHL “hypoxia sensing triad”, the FIH-HIFα CTAD “diad” is not universally conserved, being only sporadically present in simple animals, implying it evolved subsequent to the PHD enabled sensing mechanism.^16^

Extensive genetic and chemical intervention studies support the physiological importance of the HIFα-PHD-pVHL triad in animals. Indeed, the human PHDs are current therapeutic targets, with PHD inhibitors for the treatment of anemia, via HIF mediated upregulation of erythropoietin, being in late stage clinical trials.^17^,^18^ In humans, there are three PHDs (PHD1-3) and three HIFα paralogues, but only one FIH. Animal studies imply that PHD2, which, unlike PHD1 and 3, contains a highly conserved Myeloid, Nervy, and DEAF-1 (MYND) finger domain, is the most important of the three mammalian PHDs from a physiological perspective.^19^,^20^ Unlike humans and other mammals, there is only one PHD present in (most) lower organisms, which is more similar to PHD2 than PHD1 or PHD3.^16^

The human HIF system regulates hundreds of different genes in a context and cell-type dependent manner, with emerging evidence suggesting different roles for HIF1α and HIF2α and the three PHDs.^12^,^21^–^23^ HIF1α and HIF2α each have two ODDs, the *C*-terminal and *N*-terminal (CODD and NODD), respectively, which manifest differential sensitivities to hypoxia.^12^,^24^ In contrast to the extensive studies conducted on HIF1α and HIF2α, the biological role of HIF3α, which contains a single ODD, is less understood, with a role for HIF3α splice variants in the regulation of the hypoxic response being proposed.^25^–^27^

The three human PHDs also have different ODD selectivities, with PHD1 and PHD2 accepting both NODD and CODD as substrates; however, PHD3 has a strong preference for CODD over NODD as a substrate.^21^,^22^ Biochemical studies on the PHDs are supportive of their roles as hypoxic sensors.^21^,^28^–^30^ An essential requirement for the proposed hypoxia sensing role of the PHDs is that their activity in cells is regulated by oxygen availability. Studies, both in cells and animals, support the proposed hypoxia sensing role of the PHDs,^1^–^9^ raising the question as to whether their hypoxia sensing role is manifested in specific biochemical properties. There is potential for various factors to help enable PHD activity to be limited by oxygen availability in cells, including regulation of localization, concentrations, or activities of the PHDs and HIFα isoforms. Several studies support the proposal that the kinetic properties of the PHDs are directly related to their sensing role.^21^,^28^–^30^ Strikingly, while PHD2, the most conserved of the human PHDs, binds Fe(II) and 2OG relatively tightly compared to other 2OG oxygenases, forming an unusually stable enzyme.Fe.2OG complex, the PHD2.Fe.2OG.ODD complex reacts unusually slowly with oxygen (Figure S1).^30^,^31^ Structural analyses on PHD2 have led to the proposal that its slow reaction with oxygen is related to the active site coordination chemistry, with substitution of an Fe(II) bound water by dioxygen being a limiting step in catalysis.^32^–^36^ To investigate the proposal that the apparently special kinetic properties of PHDs are a general feature of their hypoxia sensing roles, we have initiated studies on their evolution.

Bioinformatic studies suggest that the HIFα-PHD-pVHL triad only exists in animals, though homologues of the PHDs, but not apparently HIF, are present in earlier organisms.^16^,^37^,^38^ Roles for PHD homologues in early eukaryotes, ie, hydroxylation of S-phase kinase associated protein 1 (Skp1) in *Dictyostelium*,^37^ and in some bacteria, ie, hydroxylation of the Elongation Factor EF-Tu^38^ have been identified. However, while these enzymes also catalyze prolyl C-4 hydroxylation and their overall structures are clearly related to the PHDs, the available evidence is that these enzymes differ in their kinetic properties relative to the human PHDs,^37^–^39^ as is the case for the procollagen prolyl-4-hydroxylases.^21^,^28^,^40^–^46^

The extent to which there is structural conservation of the active sites between the PHDs from different animals has been unclear to date; we have reported experimental evidence for the presence of a functional HIFα-PHD-pVHL system in *Trichoplax adhaerens*, the simplest known animal (Figure S2).^16^ Interestingly, the genes encoding for *Ta*HIFα and *Ta*PHD are adjacent in the *T. adhaerens* genome, suggesting linked evolution, and *Ta*PHD activity can be limited by oxygen availability in human cells.^16^ Here we report crystallographic and biochemical studies on the single PHD present in *T. adhaerens* (*Ta*PHD, Uniprot ID: I6QVT6), the results of which reveal striking elements of structural and biochemical conservation between *Ta*PHD and human PHD2 (*Hs*PHD2) active sites, supporting oxygen binding/reaction as a key property in the oxygen sensing capability of the PHDs. The results also provide insights into the evolution of ODD binding by PHDs, and help describe how human PHDs achieve ODD selectivity.

## Results

### Steady-state kinetic studies of *Ta*PHD in comparison to *Hs*PHD2

**Figure 2:**
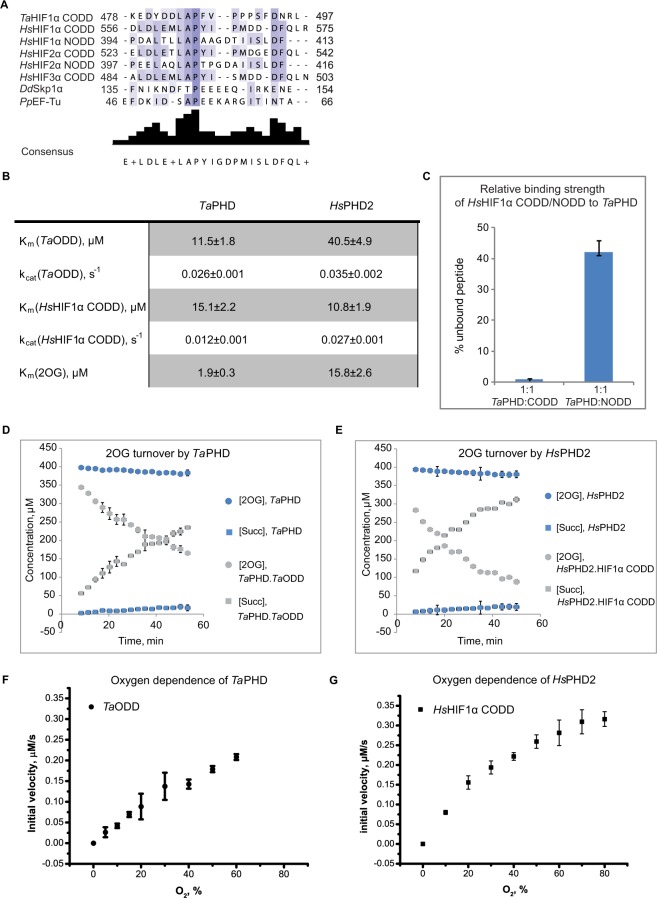
Evidence for conservation of biochemical properties between *Ta*PHD and HsPHD2. **Notes:** (**A**) Sequence alignment of *Ta*HIFa, *Hs*HIFa, and substrates of PHD-like enzymes in *Dictyostelium discoideum* (*Dd*Skp1) and *Pseudomonas putida* (*Pp*EF-Tu). (**B**) Kinetic parameters determined for *Ta*PHD and *Hs*PHD2 by MALDI-TOF-MS based assays, conditions: *Hs*PHD2 or *Ta*PHD (3.5 µM–7.0 µM), *Hs*HIF1α CODD 19mer peptide (DLDLEMLA**P**YIPMDDDFQL-NH_2_, 100 µM) or *Ta*HIFa ODD 25mer peptide (PINEKEDYDDLA**P**FVPPPSFDNRLY-NH_2_, 100 µM), (NH_4_)_2_Fe(II)(SO_4_)_2_ (50 µM), sodium L-ascorbate (4 mM) and 2OG disodium salt (300 µM) in Tris (50 mM), pH 7.5. Initial rates were determined by varying the concentrations of the respective peptide or 2OG. Peptide hydroxylation was analyzed by MALDI-MS; the apparent non-enzymatic Met oxidation was subtracted. Data were fitted with the Michaelis-Menten equation using GraphPad Prism^®^ (errors are indicated as standard deviations, n=3). (**C**) Results of 1D CLIP HSQC [^13^C]-*Hs*HIF1α CODD and [^13^C]-*Hs*HIF1α NODD displacement experiments with a [^13^C]-prolyl-labelled reporter *Hs*HIF1α CODD/NODD peptide reveal a higher binding affinity of *Ta*PHD for *Hs*HIF1α CODD over NODD (errors are indicated as standard deviations, n=3); conditions: [^13^C]-proline *Hs*HIF1α CODD/NODD (DLDLEMLA**P**YIPMDDDFQL-NH_2_/DALTLLA**P**AAGDTIISLDF-NH_2_, 50 µM), *Ta*PHD (50 µM), 2OG disodium salt (50 µM) buffered with Tris-D_11_ (50 mM), pH 7.5, in 10% D_2_O and 90% H_2_O. (**D**) Comparison of coupled and uncoupled (ie, in the absence of substrate) 2OG turnover by *Ta*PHD and (**E**) *Hs*PHD2. 2OG turnover was monitored by ^1^H CPMG NMR, conditions: *Ta*PHD or *Hs*PHD2 (20 µM), (NH_4_)_2_Fe(II)(SO_4_)_2_ (125 µM), sodium L-ascorbate (1 mM), *Hs*HIF1α CODD (500 µM) or *Ta*HIFa ODD substrate (500 µM) (where necessary), and 2OG disodium salt (400 µM), in 10% D_2_O and 90% H_2_O, Tris-D_11_ (50 mM), pH 7.5; [Succ]=Succinate. (**F**) Steady-state O_2_-dependence of *Ta*PHD and (**G**) *Hs*PHD2 (published in;^29^ conditions: 4 µM *Ta*PHD/*Hs*PHD2, 100 µM *Ta*HIFa ODD/*Hs*HIF1α CODD, (NH_4_)_2_Fe(II)(SO_4_)_2_ (50 µM), 2OG disodium salt (300 µM) and sodium L-ascorbate (4 mM) in Tris (50 mM), pH 7.5 were incubated at 37°C under different % O_2_. The extent of hydroxylation was analyzed by MALDI–ToF-MS (errors are indicated as standard deviations, n=3).

The HIF system in *T. adhaerens* is apparently markedly simpler than in humans because it contains only one PHD (*Ta*PHD), and one HIFα isoform with a single ODD (*Ta*ODD, Figure 1A).^16^ Although the sequences of human HIF1α CODD and NODD (*Hs*HIF1α CODD/NODD), and *Ta*ODD differ substantially (Figure 2A), *N*-terminally truncated *Ta*PHD (aa 64-300, *Ta*PHD^64-300^) catalyzes prolyl hydroxylation of both the *Ta*ODD 25mer and the human HIF1α CODD and HIF1α NODD 19mer substrates, with the latter being hydroxylated less efficiently.^16^ To further investigate the substrate preferences of *Ta*PHD^64–300^, its affinities for *Ta*ODD 25mer, human HIF sequences, and its co-substrate (2OG) were determined in steady-state kinetic studies and compared to the truncated human PHD isoform 2 (*Hs*PHD2^181-426^). The kinetic parameters for the catalytic domain of *Hs*PHD2^181-426^ have been shown to be similar to those of the full-length protein.^31^,^59^

K_m_ and k_cat_ values (Figure 2B) for HIF peptide substrates and 2OG were determined under optimized assay conditions described in “Experimental Methods” in Supplementary materials, with the extent of peptide hydroxylation being analyzed by Matrix-Assisted Laser Desorption/Ionization (MALDI) time-of-flight mass spectrometry (MS) (Figures 2B and S3–5). Consistent with previous reports,^16^ *Ta*PHD^64-300^ efficiently catalyzes the hydroxylation of its natural substrate *Ta*ODD 25mer and the human HIF1α CODD 19mer peptide. The *Hs*HIF1α NODD 19mer, by contrast, is not turned over above background level by *Ta*PHD^64-300^, at least under the conditions/limits of detection of the assays employed here (Figure S5). *Ta*PHD^64-300^ has a similar affinity to both these substrates, despite their differences in length, as judged by K_m_ values (K_m_(*Ta*ODD 25mer, *Ta*PHD^64-300^)=11.5±1.8 µM, K_m_(*Hs*HIF1α CODD 19mer, *Ta*PHD^64-300^)=15.1±2.2 µM). However, the catalytic efficiency of *Ta*PHD^64-300^ as judged by k_cat_ is lower with the human CODD sequence (k_cat_ (*Hs*HIF1α CODD 19mer, *Ta*PHD^64-300^)=0.012±0.001 s^−1^, k_cat_ (*Ta*ODD 25mer, *Ta*PHD^64-300^)=0.026±0.001 s^−1^). *Hs*PHD2^181-426^, by comparison, manifests a decreased affinity for the *Ta*ODD (K_m_(*Hs*HIF1α CODD 19mer, *Hs*PHD2^181-426^)=10.8±1.9 µM, K_m_(*Ta*ODD, *Hs*PHD2^181-426^)=40.5±4.9 µM). *Hs*PHD2^181-426^, however, catalyzes hydroxylation of *Hs*HIF1α CODD 19mer and *Ta*ODD 25mer with comparable efficiency k_cat_ (*Hs*HIF1α CODD 19mer, *Hs*PHD2^181-426^)=0.027±0.001 s^−1^, k_cat_ (*Ta*ODD 25mer, *Hs*PHD2^181-426^)=0.035±0.002 s^−1^).

Notably, the affinity of *Ta*PHD^64-300^ for 2OG (K_m_(2OG, *Ta*PHD^64-300^)=1.9±0.3 µM) is higher than for *Hs*PHD2^181-426^ (K_m_(2OG, *Hs*PHD2^181-426^)=15.8±2.6 µM), as judged by K_m_ comparisons. The higher affinity of 2OG for *Ta*PHD^64-300^ is in accord with the observation of formation of a stable *Ta*PHD. Fe.2OG complex, with a half-life >24 h.^16^ The kinetic parameters reported here for the affinity of *Hs*HIF1α CODD 19mer and 2OG for *Hs*PHD2^181-426^ are consistent with previous studies,^29^ K_m_(*Hs*HIF1α CODD 19mer, *Hs*PHD2^181-426^)=9±3 µM, K_m_(2OG, *Hs*PHD2^181-426^)=13±2 µM).

### *Ta*PHD binds *Hs*HIF1α NODD less efficiently than *Hs*HIF1α CODD

The observed preference of *Ta*PHD^64-300^ for *Hs*HIF1α CODD over *Hs*HIF1α NODD is in agreement with the finding of one ODD in the genome of *T. adhaerens*, which is closer in sequence to the majority of CODD, compared to NODD sequences (Figure 2A).^16^ While a NODD-like sequence is present in all vertebrates, NODD is apparently not present in some non-vertebrates, eg, Placozoa and Cnidaria, suggesting that the CODD sequence might have evolved prior to that of NODD.^16^ Notably, like *Ta*PHD^64-300^, one of the human PHD isoforms, *Hs*PHD3, is highly selective for *Hs*HIF1α/2α CODD over *Hs*HIF1α/2α NODD.^1^,^21^,^47^

To more directly investigate the extent to which *Ta*PHD^64-300^ can bind *Hs*HIF1α NODD and CODD, we performed 1D CLIP HSQC NMR binding studies with *Hs*HIF1α CODD and *Hs*HIF1α NODD peptides labeled with [^13^C] at the target prolyl residues which undergo hydroxylation during catalysis (Figure 2C). The [^13^C]-labeled “reporter” *Hs*HIF1α CODD/NODD peptides were incubated with Zn(II) and 2OG in the presence and absence of *Ta*PHD^64-300^ (1:1 enzyme:ODD, for conditions see “Experimental methods” in Supplementary materials). The decrease of the reporter signal upon binding to the enzyme was measured to investigate the relative binding strength (Figure 2C). Consistent with the kinetic studies, the NMR results reveal that *Ta*PHD^64-300^ strongly discriminates between binding of *Hs*HIF1α CODD and *Hs*HIF1α NODD, aŝ40% of the *Hs*HIF1α NODD peptide remained unbound, while *Hs*HIF1α CODD was apparently completely bound to *Ta*PHD^64-300^, showing that *Ta*PHD^64-300^ binds *Hs*HIF1α CODD better than *Hs*HIF1α NODD.

### Monitoring the coupled and uncoupled 2OG turnover of *Ta*PHD

2OG dependent oxygenases catalyze the uncoupled turnover of 2OG to succinate and CO_2_ in the absence of their prime substrate to varying extents;^48^ in the case of *Hs*PHD2, it has been shown that 2OG decarboxylation is strongly coupled to CODD hydroxylation.^31^ To directly compare the extent of uncoupled turnover in *Ta*PHD^64-300^ and *Hs*PHD2^181-426^, succinate formation and 2OG depletion were monitored by ^1^H-NMR^49^ in the presence and absence of their respective natural substrates (Figure 2D and E). The results demonstrate that the rate of uncoupled 2OG turnover is very low for both *Ta*PHD^64-300^ and *Hs*PHD2^181-426^; 2OG decarboxylation is strongly increased in presence of substrate, implying conservation of (at least some) kinetic properties between *Hs*PHD2 and *Ta*PHD. Overall, these results support the proposal that the strong binding of Fe(II) and 2OG, and the formation of a stable *Hs*PHD2.Fe.2OG complex in the absence of *Hs*HIFα, are conserved properties of the PHDs.

### Oxygen dependence of *Ta*PHD

In order for the PHDs to act as hypoxia sensors in cells, their activity must be limited by oxygen availability. This role is proposed to be reflected in the slow reaction of (at least) the *Hs*PHD2.CODD/NODD.Fe.2OG complexes with oxygen.^29^,^31^ It is also proposed to manifest in the unusually high K_m_ value (for 2OG oxygenases) of *Hs*PHD2 for oxygen (K_m_(O_2_, *Hs*PHD2) >400 µM).^21^,^28^,^29^,^50^ In order to study the O_2_ dependence of the *Ta*PHD-catalyzed reaction, steady-state kinetics, using reported experimental conditions for *Hs*PHD2;^29^ were conducted. *Ta*PHD^64-300^ was reacted with its peptidic substrate in sealed glass-vials under varied oxygen concentrations,^29^ the initial rates of *Ta*ODD 25mer hydroxylation were determined by MALDI–TOF-MS (Figure 2F and G). No saturation of O_2_ was reached for the *Ta*PHD^64-300^-catalyzed reaction with *Ta*ODD 25mer at 60% O_2_ level, resulting in a K_m_ (O_2_, *Ta*PHD^64-300^) >400 µM. This result suggests that *Ta*PHD has the potential to react to small changes in atmospheric O_2_ partial pressure and could act as an effective oxygen sensor, indicating that the core biochemical features of *Hs*PHD2 in the human oxygen sensing machinery may well be conserved in *Ta*PHD. It has been reported that *Hs*PHD2 reacts relatively slowly with oxygen, compared to other 2OG oxygenases as determined by stopped flow/UV-visible spectroscopy experiments.^29^,^31^ It was not possible to conduct a similar study on *Ta*PHD for practical reasons (ie, neither *Ta*PHD^64-300^, nor other tested constructs *Ta*PHD^1-300^ and *Ta*PHD^79-300^ could reach a sufficiently high protein concentration necessary for stopped-flow assays due to aggregation).

### Crystallization of *N*-terminally truncated *T. adhaerens* PHD (*Ta*PHD^64-300^)

In order to investigate the extent to which the structural features proposed to be necessary for oxygen sensing are conserved between *Hs*PHD2 and *Ta*PHD, we attempted to obtain a crystal structure of *Ta*PHD in complex with Mn(II), a non-reactive substitute for Fe(II), using a *Ta*PHD construct (aa 64-300, *Ta*PHD^64-300^, MW=29.2 kDa) lacking the *N*-terminal MYND domain, which could be produced efficiently in *Escherichia coli*. The shorter construct (*Ta*PHD^64-300^) yielded crystals that diffracted to 1.2 Å resolution (Table 1). The structure was solved by molecular replacement, using *Hs*PHD2 (PDB: 2G19),^32^ as a search model. To gain insight into the substrate-binding mode of *Ta*PHD, we then co-crystallized *Ta*PHD^64-300^ with a 21mer peptide fragment of its substrate, the *Ta*HIFα ODD (*Ta*ODD, residues E477-L497), Mn(II) and *N*-oxalylglycine (NOG), a close 2OG analog. The *Ta*PHD.*Ta*ODD.Mn.NOG structure (Table 1) was solved to a resolution of 1.3 Å by molecular replacement, using the structure without *Ta*ODD as a search model. The substrate-bound and unbound forms of *Ta*PHD crystallized in the *P*1 and *P*2_1_ space groups, respectively, reflecting different packing, possibly relating to structural changes in *Ta*PHD induced upon substrate binding.

### Overall structure of *Ta*PHD

**Figure 3:**
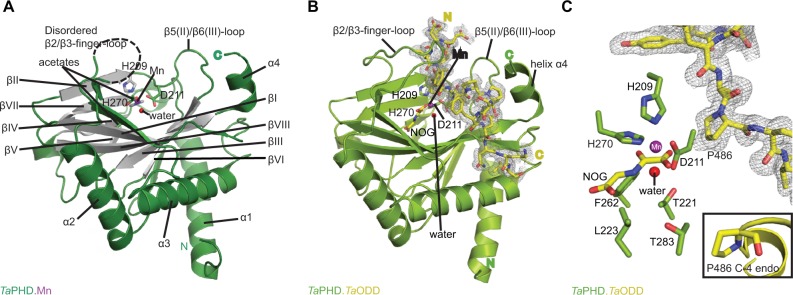
Views from crystal structures of the *Trichoplax adhaerens* prolyl hydroxylases (PHD) without substrate bound (*Ta*PHD) and in complex with a fragment of its substrate (*Ta*PHD.*Ta*ODD). **Notes:** (**A**) Secondary structural elements in *Ta*PHD comprise four α helices and ten β strands, eight of which form the double-stranded β-helix core fold (DSBH, gray, Roman numerals I–VIII). (**B**) Overall binding mode of *Ta*ODD to *Ta*PHD in the *Ta*PHD.*Ta*ODD complex structure showing the 2F_o_-F_c_ electron density map for the peptidic substrate (gray mesh, contoured to 1.0 σ). (**C**) Active site close-up of *Ta*PHD.*Ta*ODD reveals that the P486 *Ta*ODD C-4 methylene adopts an *endo*-conformation (2F_o-_F_c_ density, gray mesh, contoured to 1.0 σ). The metal ion (manganese substituting for iron, purple sphere) is octahedrally coordinated by a triad of residues (H209*_Ta_*_PHD_, D211*_Ta_*_PHD_, and H270*_Ta_*_PHD_), *N*-oxalylglycine (NOG), and a water molecule (W1, red sphere). The stable metal-water coordination observed here is conserved in *Hs*PHD2,^32^,^34^ where it is proposed to enable the oxygen sensing ability in *Hs*PHD2.

The structure of *Ta*PHD (Figure 3A) contains the conserved double-stranded β-helix core-fold (DSBH, or “jelly roll”-motif), which is present in all characterized 2OG dependent oxygenases, including the human PHDs.^15^ The DSBH is comprised of the major (βI, βVIII, βIII, βVI) and minor (βII, βVII, βIV, βV) β-sheets between which the metal and 2OG binding sites are sandwiched. As with *Hs*PHD2, the major β-sheet core is stabilized by three α-helices (α1–3). The overall folds of the substrate-unbound *Ta*PHD (*Ta*PHD. Mn) and the substrate-bound *Ta*PHD.*Ta*ODD complex are very similar (Figure 3A and B, RMSD for all Cα=0.15 Å). Eighteen residues of the 21mer *Ta*ODD peptide (A480*_Ta_*_ODD-_L497*_Ta_*_ODD_, Figure 3B) are visible in the *Ta*PHD.*Ta*ODD structure.

**Figure 4:**
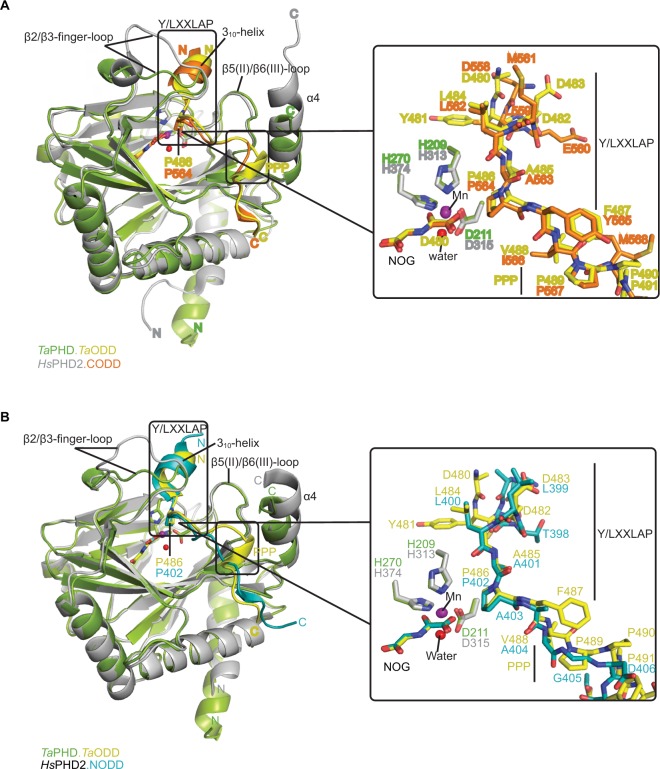
Comparison of ODD binding modes by the *T. adhaerens* and human HIFα PHD for the *Ta*PHD.*Ta*ODD, *Hs*PHD2.CODD and *Hs*PHD2.NODD substrate structures. **Notes:** (**A**) Superimposition of structural views of *Ta*PHD.*Ta*ODD with *Hs*PHD2.CODD (PDB: 3HQR) and (**B**) *Ta*PHD.*Ta*ODD with *Hs*PHD2.NODD (PDB: 5L9V) reveals major differences between the structures in the PHD flexible β2/β3 finger-loop and PHD *C*-terminal substrate binding interfaces. Notably, the Pro-Pro-Pro motif in *Ta*ODD (PPP motif, P489-491*_Ta_*_ODD_) adopts a helical bend, which aligns poorly with *Hs*HIF1α CODD and, particularly, with *Hs*HIF1α NODD. **Abbreviations:** *T. adhaerens*, *Trichoplax adhaerens*; ODD, oxygen dependent degradation domain.

**Figure 5:**
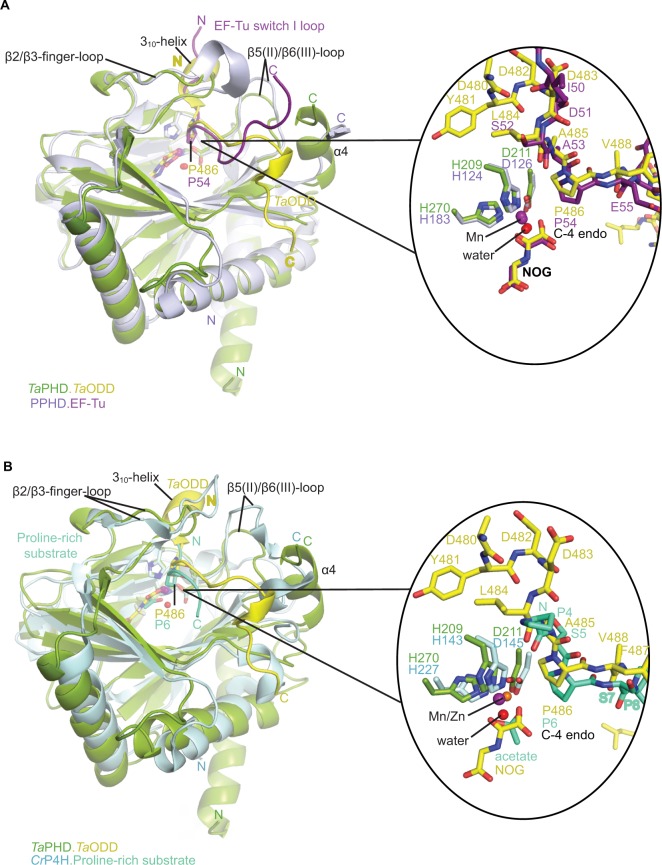
Comparison of substrate binding modes by *Ta*PHD, *Cr*P4H and *Pseudomonas putida* PPHD. **Notes:** Overall superimposition and active site close-up of the *Ta*PHD.*Ta*ODD complex with (**A**) *P. putida* PPHD (PDB: 4IW3) and (**B**) *Cr*P4H (PDB: 3GZE) in complex with a proline rich peptidic substrate reveals the conservation of the substrate-binding mode involving the conformationally flexbile β2/β3-finger-loop (in all cases) and the *C*-terminal α4-helix (in case of *Ta*PHD and PPHD).

**Figure 6:**
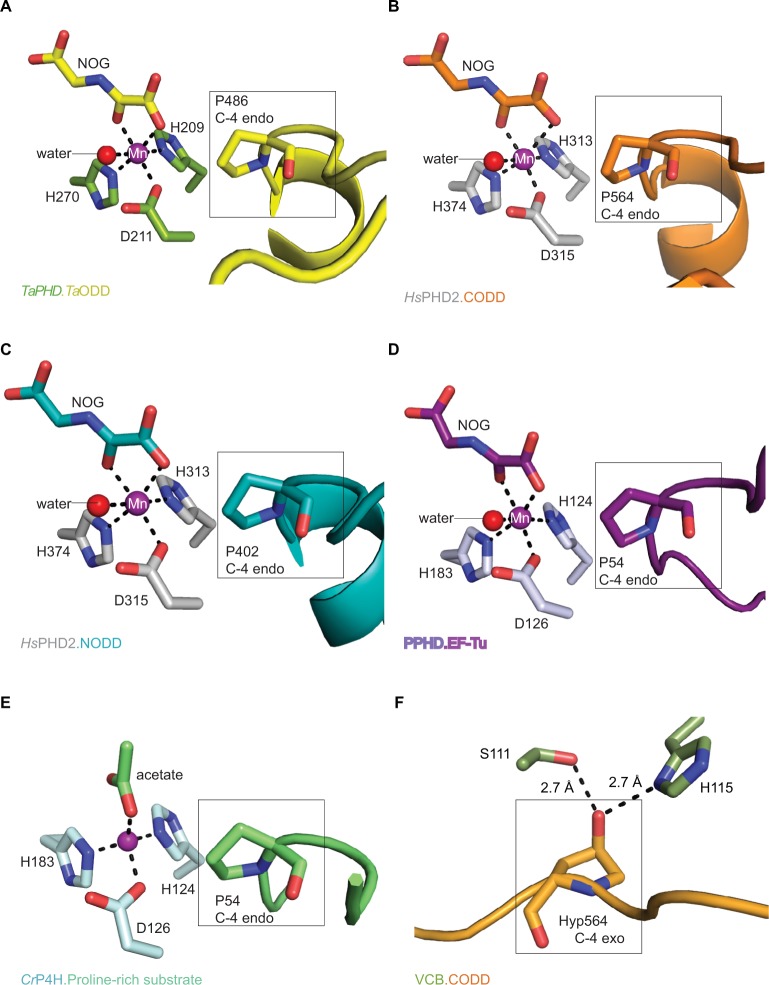
Active site metal region details and comparison of the conformations of the target prolyl-residues in the enzyme-substrate complex structures of *Ta*PHD, *Hs*PHD2, PPHD, and *Cr*P4H. **Notes:** (**A**) *Ta*PHD.*Ta*ODD, (**B**) *Hs*PHD2.CODD (PDB: 3HQR,^34^), (**C**) *Hs*PHD2.NODD (PDB: 5L9V,^34^), (**D**) PPHD.EF-Tu, (PDB: 4IW3,^38^) and (**E**) *Cr*P4H.proline-rich substrate (PDB: 3GZE,^66^) complex structures. Note that, in all the enzyme-substrate complexes, the proline C-4- methylene, that is hydroxylated, adopts the *endo*-conformation. (**F**) By contrast, Hyp564 in *Hs*HIF1α CODD adopts the C-4 *exo*-conformation when bound to the VCB complex (PDB: 1LM8,^55^–^57^). In (**A** and **D**), *N*-oxalylglycine (NOG) acts as a 2OG analog. Note that the Zn (substituting for Fe) in the *Cr*P4H active site is tetrahedrally coordinated, with an acetate binding instead of the 2OG co-substrate. The structures reveal conserved orientations of the “target” proline residues, which in each case, adopt a C-4 *endo*-conformation.^55^ Note that the metal bound water present in the *Ta*PHD (and *Hs*PHD2/PPHD) substrate complex structures is not observed in the *Cr*P4H.(Ser-Pro)_5_ structure.

Clear differences between the *Ta*PHD.Mn and *Ta*PHD. *Ta*ODD structures manifest in the mobile “β2/β3-finger-loop”,^32^–^34^ which is disordered in the substrate-unbound *Ta*PHD structure (missing residues Q137*_Ta_*_PHD_-R146*_Ta_*_PHD_). In the substrate-bound structure, however, the β2/β3-finger-loop is ordered and makes extensive contacts with the substrate. By contrast, the β7(VI)/β8(V)-loop residue N245*_Ta_*_PHD_ is not observed in the *Ta*PHD.*Ta*ODD structure, unlike in the substrate-unbound *Ta*PHD.Mn structure, in which the electron density of the complete β7(VI)/β8(V)-loop is apparent (Figures 3–6, S4 and S6).

### Active site of *Ta*PHD

The active site pocket of *Ta*PHD, positioned at one end of the DSBH, deeply embeds the metal ion (Mn, substituting for Fe) and the co-substrate (NOG, substituting for 2OG, Figure 3B and C). The highly buried nature of the 2OG binding site likely contributes to the formation of a stable *Ta*PHD.Fe.2OG complex, as reflected in the observed low rate of uncoupled 2OG turnover by *Ta*PHD (Figures 2D and 3B).

In the *Ta*PHD.Mn structure without *Ta*ODD, the manganese ion is octahedrally coordinated by a conserved triad of residues (H209*_Ta_*_PHD_, D211*_Ta_*_PHD_, and H270*_Ta_*_PHD_), an acetate ion, and two water molecules.^48^,^51^ Deep within the 2OG binding pocket an additional acetate ion forms a salt bridge with R279*_Ta_*_PHD_, apparently mimicking 2OG C5 carboxylate binding (Figure 3A).

The 6-coordinate state of the metal is retained in the *Ta*PHD.*Ta*ODD complex. Metal-ion binding involves the highly conserved triad of metal-coordinating residues, and an ordered, metal-bound water molecule (W1) that is, in addition to the metal coordination, stabilized via hydrogen bonding with the metal-ligating side chain of D211*_Ta_*_PHD_ (Figures 3C and 6). Electron density corresponding to the 2OG analog inhibitor, NOG, is clearly visible in the *Ta*PHD. *Ta*ODD complex structure. NOG coordinates to the metal ion in a bidentate manner and interacts with R279*_Ta_*_PHD_ by its C5 carboxylate. The 1-carboxylate of NOG is positioned at the coordination site closest to the hydroxylation target, P486*_Ta_*_ODD_, making the remaining metal coordination site, occupied by water W1, less accessible, and thus apparently hindering oxygen binding to the metal ion (Figure 6).^33^,^34^

Crystal structures of the human PHD isoform 2 (*Hs*PHD2) in complex with its substrates *Hs*HIF1α CODD (referred to as *Hs*PHD2.CODD, PDB: 3HQR^34^) and *Hs*HIF1α NODD (referred to as *Hs*PHD2.NODD, PDB: 5L9V^33^) have been reported (Figure 4). The overall active site arrangement observed in the *Ta*PHD.*Ta*ODD complex is very similar to those observed in the *Hs*PHD2.CODD/NODD complexes (Figures 4–6,^33^,^34^). Such conservation includes the octahedral coordination of the metal ion by H313*_Hs_*_PHD_*_2_*, D315*_Hs_*_PHD_*_2_*, and H374*_Hs_*_PHD_*_2_*, NOG, and a metal-bound water molecule^33^,^34^,^52^. The observation of a well-ordered metal bound water in both *Ta*PHD and *Hs*PHD2 structures is important from a hypoxia sensing perspective, because displacement of this water from the metal is required for oxygen to bind, and is proposed to be rate limiting in *Hs*PHD2 catalysis.^32^,^34^,^53^ The buried nature of the metal, the 2OG analog NOG, and the associated ligating water molecule, as observed both in *Ta*PHD and *Hs*PHD2, are likely substantially responsible for the unusual stability of the *Hs*PHD2.Fe.2OG complex,^30^ compared to other 2OG oxygenases such as FIH, which is active at lower oxygen concentrations.^12^,^13^,^54^

Taken together, the observation of conserved active site arrangements in the *Ta*PHD.*Ta*ODD and *Hs*PHD2.CODD crystal structures correlates well with the steady-state kinetic parameters determined for 2OG and oxygen, imply ing that core features necessary for the oxygen-sensing role of the enzymes are conserved between *Ta*PHD and *Hs*PHD2.

### Comparison of ODD binding modes for *Ta*PHD and *Hs*PHD2

The main substrate binding elements in the *Ta*PHD.*Ta*ODD complex structure, as in *Hs*PHD2, comprise: the active site containing groove, the β2/β3-finger-loop, and the *C*-terminal α4-helix (Figure 3B). The *Ta*ODD peptide binds to *Ta*PHD in an extended form; notably, the residues *N*-terminal to the target proline form a 3_10_-helix. The target proline in *Ta*ODD and its *N*-terminally flanking residues form a YXXLAP motif, which differs by one residue (L->Y) compared to the consensus LXXLAP prolyl-hydroxylation site(s) present in all human, and many, but not all animal, HIFα proteins.^16^

Binding and positioning of the hydroxylation target P486*_Ta_*_ODD_ directly adjacent to the metal in the active site of *Ta*PHD is highly similar to that in the *Hs*PHD2.CODD and *Hs*PHD2.NODD complex structures (Figure 4A and B). Residues Q137*_Ta_*_PHD_, L138*_Ta_*_PHD_, and A139*_Ta_*_PHD_ in the β2/β3-finger-loop region (corresponding to Q239*_Hs_*_PHD_*_2_*, L240*_Hs_*_PHD_*_2_*, and V241*_Hs_*_PHD2_) form H-bonds and hydrophobic contacts with *Ta*ODD residues P486*_Ta_*_ODD_*_,_* F487*_Ta_*_ODD_*_,_* A485*_Ta_*_ODD_ (P564*_Hs_*_HIF1α_ *_C_*_ODD_, A563*_Hs_*_HIF1α_ *_C_*_ODD_, Y565*_Hs_*_HIF1α_ *_C_*_ODD_ and P402*_Hs_*_HIF1α_ *_N_*_ODD_, A401*_Hs_*_HIF1α_ *_N_*_ODD_, A403*_Hs_*_HIF1α_ *_N_*_ODD_, respectively). The hydroxylation target proline is apparently further positioned via polar interactions with βII(β5)/βIII(β6) residues Y206*_Ta_*_PHD_ and R218*_Ta_*_PHD_ (Y310*_Hs_*_PHD_*_2_* and R322*_Hs_*_PHD_*_2_*), and hydrophobic contacts are formed to residue W285*_Ta_*_PHD_ (W389*_Hs_*_PHD_*_2_*) in the *C*-terminal strand βVIII(β11), (Table S1, Figure S4).

As observed in the *Hs*PHD2.CODD/NODD structures, the substrate proline P486*_Ta_*_ODD_ pyrrolidine ring adopts a C-4 *endo*-conformation, with the 4*R* C-H bond that is cleaved during hydroxylation, being positioned close to the metal center (distance: 4.5 Å, Figures 3C and 6,^33^,^34^); indeed, the conformations of the substrate target prolyl rings at the *Ta*PHD and *Hs*PHD2 active sites are nearly identical (Figure 4A and B). Upon hydroxylation by *Hs*PHD2, the conformation of the pyrrolidine ring in CODD is proposed to switch from C-4 *endo* to C-4 *exo*^55^ (Figure 6). When bound to pVHL, the C-4 hydroxylated proline adopts the C-4 *exo* conformation as favored by stereoelectronic theory (the gauche effect);^55^–^58^ a role for this conformational change in promoting product release from *Hs*PHD2 has been suggested.^34^

In the human PHDs, the β2/β3-finger-loop plays a significant role in substrate recognition and in determining NODD/CODD selectivity.^33^,^34^,^59^ The β2/β3-finger-loop is partially disordered without its substrate bound, likely reflecting flexibility relative to the enzyme-substrate complex.^33^ By contrast, in both the *Hs*PHD2.CODD and *Hs*PHD2.NODD structures, the β2/β3-loop residues fold to enclose the LXXLAP substrate motif at the active site (Figure 4A and B). Although, the β2/β3-finger–loop in the *Ta*PHD.*Ta*ODD complex also constitutes one of the major substrate interaction sites, it is two residues shorter than in *Hs*PHD2 between N141*_Ta_*_PHD_-V142*_Ta_*_PHD_ (Figures S4 and 6). As a consequence, and by contrast to *Hs*PHD2,^33^ the β2/β3- finger-loop in the *Ta*PHD.*Ta*ODD structure is more condensed and stabilized by intramolecular interactions between loop residues, including backbone-backbone H-bonds and salt bridges between side chains (Figure S3). This structural difference might reflect the fact that there is only a single HIFα isoform with one ODD in *T. adhaerens*, which contrasts with the situation in humans where there are three HIFα isoform substrates; with both a NODD and a CODD in case of HIF1α and HIF2α (note other *Hs*PHD substrates have also been reported).^60^–^65^ It is thus possible that binding of multiple HIF substrates to *Hs*PHD2^33^,^34^ requires a higher degree of flexibility in the β2/β3-finger-loop, compared to the apparently more rigid β2/β3-finger-loop in *Ta*PHD.

In both the *Ta*PHD.*Ta*ODD and *Hs*PHD2.CODD^34^ structures, a salt-bridge between an arginine residue in the *C*-terminal α4-helix and an aspartate residue in the *C*-terminus of the respective peptide (R292*_Ta_*_PHD_/D494*_Ta_*_ODD_*_,_* R396*_Hs_*_PHD_*_2_*/D571*_Hs_*_HIF1α_ *_C_*_ODD_) is formed (Figure S4). However, this salt-bridge is not present in the *Hs*PHD2.NODD complex structure, where the *Hs*HIF1α NODD peptide is positioned further from the *C*-terminal helix and interacts with the *C*-terminal region of *Hs*PHD2 via hydrophobic contacts. Studies with the *Ta*ODD and *Hs*PHD2 variants, including with *Hs*PHD2 P317R*_Hs_*_PHD_*_2_* and R396T*_Hs_*_PHD_*_2_*, which are selective for CODD or NODD, respectively, support the proposal of a similar binding mode for *Ta*ODD and *Hs*HIF1α CODD/NODD to *Hs*PHD2 (Figure S4).

By contrast with *Hs*HIF1α CODD/NODD peptides, the *Ta*ODD adopts a short 3_10_-helix near its Pro-Pro-Pro motif (P489*_Ta_*_ODD_-491*_Ta_*_ODD_), which is *C*-terminal to the target proline P486*_Ta_*_ODD_ residue (Figure 4A and B). Notably, the Pro-Pro-Pro motif and the adjacent 3_10_-helix constitute the interaction surface with the *C*-terminal α4-helix of *Ta*PHD (Figure 4A and B). In the *Ta*PHD.*Ta*ODD complex structure, the *Ta*ODD is positioned particularly close to the α4-helix of *Ta*PHD, compared to both *Hs*HIF1α CODD and *Hs*PHD2.NODD, whereas the latter is positioned even more distant from the *C*-terminus of *Hs*PHD2 (Figure 4A and B).

### Comparison of HIF- and non-HIF prolyl-4-hydroxylase structures

Crystal structures of the enzyme-substrate complex of a pro-lyl-4-hydroxylase from *Chlamydomas reinhardtii* (*Cr*P4H), a member of the collagen-prolyl-4-hydroxylase-subfamily,^66^ and a *Pseudomonas putida* prolyl hydroxylase (PPHD), a clear prokaryotic PHD homologue, have been reported.^38^,^66^ Both *Cr*P4H and PPHD act on non-HIFα substrates, ie (for consistency), *Cr*P4H catalyzes the hydroxylation of a proline residue P6 in a proline-rich (Ser-Pro)_5_ substrate (PDB: 3GZE,^66^), and PPHD hydroxylates a proline residue (P54) in the translation elongation factor EF-Tu (PDB: 4IW3,^38^) (Figure 5).

Structure based sequence alignment of *Ta*PHD with the human PHD isoforms, *Chlamydomonas reinhardtii* (*Cr*P4H) and *Pseudomonas putida* (PPHD) was performed (Figure S2). Consistent with its essential role in catalysis, the 2OG-oxygenase domain (E90*_Ta_*_PHD_-F287*_Ta_*_PHD_) manifests the highest degree of sequence conservation among the HIF-and non-HIF-prolyl-4-hydroxylases. The *N*-terminal region is less well conserved, but like *Hs*PHD2, *Ta*PHD contains a conserved cysteine-rich MYND-type zinc finger domain. The function of the *N*-terminal domain of the PHDs is not fully understood; a role for the MYND domain in the interaction with heat shock protein 90 (HSP90), co-chaperones p23, and FKBP38 has been proposed.^67^

Analysis of the structural conservation between *Ta*PHD and *Hs*PHD2 (Figure S4, Table S2) reveals high overall similarity (RMSD for all Cα of *Ta*PHD_C73-Q297_.*Ta*ODD -*Hs*PHD2_Q184-K408_.CODD=0.54 Å, and RMSD for all Cα of *Ta*PHD_C73-Q297_.*Ta*ODD - *Hs*PHD2_P189-Y403_.NODD=0.39 Å). Comparisons with the other prolyl-4-hydroxylases imply that *Ta*PHD shares a stronger structural conservation with PPHD (RMSD for all Cα of *Ta*PHD_C73-Q297_.*Ta*ODD - PPHD_H7-F207_. EF-Tu=0.92 Å), than with *Cr*P4H (RMSD for all Cα of *Ta*PHD_C73-Q297_.*Ta*ODD - *Cr*P4H_W38-G250_.(Ser-Pro)_5_=2.20 Å), suggesting a closer evolutionary relationship between HIF hydroxylases and PPHD than to the algal prolyl-4-hydroxylase *Cr*P4H (Figure 4). The structural similarity between PPHD and *Ta*PHD is higher than between PPHD and *Hs*PHD2 (RMSD for all Cα of *Hs*PHD2_Q184-K408_.CODD - PPHD_H7-F207_.EF-Tu=1.57 Å) (Figures 4 and 5).

In addition to their overall structural conservation, the prolyl-4-hydroxylases adopt strikingly similar substrate-binding features (Figures 4–6 and S5). The *Ta*ODD, *Hs*HIF1α CODD/NODD, EF-Tu, and the proline-rich (Ser-Pro)_5_ substrate backbones are all oriented in the same N→C direction across the active site (Figure 5A and B). *Ta*ODD, *Hs*HIF1α CODD, and *Hs*HIF1α NODD all form a partial helical structure (3_10_-helix), when bound to enzyme (Figure 4A and B), while isolated *Hs*HIF1α CODD/NODD are predicted to be disordered in solution.^33^,^34^ By contrast, the EF-Tu switch loop retains loop secondary structure upon binding to PPHD, albeit after a large conformational change.^38^

Notably, the PHDs all employ a flexible β2/β3-finger-loop (G136*_Ta_*_PHD_-D150*_Ta_*_PHD_, S77*_Cr_*_P4H_-S95*_Cr_*_P4H_, A53_PPHD_-D70_PPHD_) that undergoes a major conformational change upon substrate binding and helps to enclose the target proline of the substrate in the active site cleft (Figures 5, 6 and S4).^33^,^34^ In *Ta*PHD, *Hs*PHD2 and PPHD, the *C*-terminus interacts with the substrate; it is unknown if this is the case for *Cr*P4H, since the shorter peptide substrate used in the *Cr*P4H.(Ser-Pro)_5_ structure does not appear to reach the *C*-terminus of the enzyme.^66^

### Conservation of proline-ring conformation

Comparison of the binding modes of the prolyl-4-hydroxylase substrates in the immediate active sites reveals a high degree of conservation (Figures 5A, B and 6). The substrates all manifest a similar orientation toward the active site residues and the metal center, and the target prolyl residues all adopt the C-4 *endo*-conformation (Figure 5A and B).^55^ Similarly to the HIF prolyl-4-hydroxylases *Ta*PHD and *Hs*PHD2, PPHD and *Cr*P4H both employ a HXD…H motif for metal-binding in the active site (H143*_Cr_*_P4H_, D145*_Cr_*_P4H_, H227*_Cr_*_P4H_, and H124_PPHD_, D126_PPHD_, H183_PPHD_).^38^,^66^ In the PPHD crystal structure, the metal ion is octahedrally coordinated and one of the coordination sites is occupied by a water molecule (Figure 6), implying evolutionary conservation in the active site arrangement between the HIF prolyl-4-hydroxylases in animals and the bacterial non-HIF prolyl-4-hydroxylase PPHD. By contrast, in the *Cr*P4H crystal structure,^66^ the metal ion is tetrahedrally coordinated; however, in this structure, binding of a water molecule to the metal is apparently blocked by an acetate ion (Figure 6E), and hence, at least in this detail, it may not be fully representative of the solution structure.

## Discussion

It has been proposed that the ferrous iron dependent oxygenases may have evolved as a response to the advent of photosynthetically produced oxygen, possibly from precursors that used relatively bioavailable iron (comparable to eg, zinc) ions, predominantly in non-redox processes.^68^,^69^ The widespread occurrence of 2OG oxygenases in prokaryotes, coupled with their close relationship with two TCA cycle intermediates (2OG and succinate), suggests they may have early origins. The discovery that 2OG oxygenases play key roles in hypoxia sensing in higher animals was therefore of interest from a broad evolutionary perspective. We are interested in exploring the biological distribution of hypoxia sensing mechanisms, as well as 2OG oxygenases and related enzymes. We have found that there is a functional HIFα-PHD-pVHL triad in the simplest animal, *T. adhaerens*.^16^ Although bioinformatics and limited experimental analyses have not provided evidence for HIF transcription factors beyond animals,^16^ PHD-like enzymes are much more widely distributed (Figure S2).^16^,^37^,^38^

The PHDs are related to the pro-collagen prolyl hydroxy-lases and are part of a structurally distinct 2OG oxygenase subfamily; the PH VIII subfamily^15^ which includes other PHDs of biological interest. In the unicellular eukaryote *Dictyostelium discoideum*, a cytoplasmic PHD homologue, which is proposed to be involved in hypoxic responses, catalyzes hydroxylation of a proline-residue in Skp1, inducing further post-translational modification of Skp1 with a pentasaccharide group that is linked via the hydroxyproline residue alcohol.^37^,^70^ Recent studies have shown that PHD homologues also exist in bacteria, where a PHD present in *Pseudomonas* species catalyzes hydroxylation of the ribosome associated protein EF-Tu.^38^

Central to the hypoxia sensing ability of the PHDs is the capacity for their hydroxylase activity in many cell types to be limited by oxygen availability.^12^,^29^ It is proposed that this is in substantial part due to the slow reaction of oxygen with the active site Fe(II) of the PHDs.^31^ By contrast there is evidence that the human PHDs are (at least normally) relatively less sensitive to changes in Fe(II) or 2OG availability, consistent with the proposed specialized role for them as hypoxia sensors.^30^

The question then arises as to what extent are these apparently special properties of the human PHDs (especially *Hs*PHD2) conserved in the PHDs/PHD-like enzymes present in animals and beyond. The results presented here provide evidence that the apparently unusual kinetic properties of *Hs*PHD2 are conserved in *Ta*PHD (Figure S7), likely as a consequence of the conservation of key structural features.

The overall folds of the catalytic domains of *Ta*PHD and the *Hs*PHD2 are very similar, including with respect to the elements involved in substrate binding, in particular the conformationally mobile β2/β3-finger-loop^33^ and the *C*-terminal helix (Figures 5 and 6). Detailed comparison of the PHD-substrate interactions reveals that the active site region interactions between *Ta*PHD and *Ta*ODD more closely resemble those between *Hs*PHD2 and *Hs*HIF1α CODD, rather than those of *Hs*PHD2 and *Hs*HIF1α NODD (Figures 4 and 6). Thus, our results support the proposal that the PHD2/CODD-type ODD couple evolved first and is likely the most important of the PHD/ODD interactions in higher animals where there are multiple PHDs and ODDs.^16^ Given the likely relatively rigid nature of the Pro-Pro-Pro motif in *Ta*ODD, it also appears that *Ta*ODD binds to *Ta*PHD in a less flexible manner than does *Hs*HIF1α NODD to *Hs*PHD2, and to a lesser extent, *Hs*HIF1α CODD (Figure 4). Although solution studies are required to validate this proposal, these differences may further reflect the evolved roles of the *Hs*PHDs (and likely those in other higher animals) in accepting multiple substrates (NODDs/CODDs of HIFα isoforms), and maybe, non-HIF substrates^60^–^65^ compared to *Ta*PHD. The conservation in overall fold is also the case with respect to the prokaryotic PHD, PPHD, which acts on the highly abundant globular GTP-utilizing protein EF-Tu,^38^ rather than the disordered ODD regions of HIFα (Figure 5). (A role of PPHD in hypoxia sensing, if any, has yet to be identified).

Crucially, the structural elements which enable the unusually slow reaction of *Hs*PHD2 with oxygen,^31^,^34^ (as well as tight 2OG/Fe(II) binding and low substrate uncoupled turnover), appear to be conserved in *Ta*PHD as observed crystallographically and manifested in the available kinetic studies (Figures 2 and 6). Since humans and *T. adhaerens* are at near opposite ends of animal evolution, it seems possible that hypoxia sensing PHD homologues in organisms between them will have similar kinetic properties. Interestingly, *Ta*PHD has a higher affinity for 2OG than *Hs*PHD2 as observed by both K_m_ comparisons and NMR binding studies. The difference in 2OG K_m_ may reflect different 2OG metabolism in *T. adhaerens* and humans. Further, like *Hs*PHD2, *Ta*PHD forms an unusually stable *Ta*PHD.Fe.2OG complex (half-life >24 h), consistent with both enzymes being special-ized to preferentially respond to changes in oxygen, rather than 2OG/Fe(II), availability.^11^ Our overall results suggest that the evolution of the HIFα (with a *Ta*ODD/CODD type ODD)-PHD2-pVHL triad, may have been an important step in evolution of animals. A key element of this triad is a PHD2-type enzyme with appropriate kinetic properties, enabling it to “focus” on hypoxia sensing. The results also suggest that searching for appropriate kinetic and structural properties may be a general approach in helping to identify candidate (hypoxia) sensing enzymes.

## Data availability

The *Ta*PHD.Mn and *Ta*PHD.*Ta*ODD structures are deposited in the RCSB PBD: entries 6EY1 and 6F0W, respectively.

## Figures and Tables

**Table 1:** Data collection and refinement statistics

	*Ta*PHD.Mn (PDB: 6EYI)	*Ta*PHD.*Ta*ODD (PDB: 6F0W)

Wavelength (Å)	0.97930	0.97950
Resolution range (Å)^a^	38.39–1.20 (1.24–1.20)	30.49–1.30 (1.35–1.30)
Space group	*P*2_1_	*P*1
Unit cell (a b c, α β γ)	40.58 59.41 51.20, 90 100.74 90	40.84 41.32 42.08, 114.06 95.67 103.69
Total reflections	640,269	377,792
Unique reflections^a^	74,236 (6949)	55,424 (5243)
Multiplicity^a^	8.6 (4.9)	6.8 (4.1)
Completeness (%)^a^	99.24 (93.39)	94.83 (89.72)
Mean *I/*σ*(I)*^a^	25.9 (2.0)	14.5 (2.6)
Wilson B-factor	13.06	13.89
*R_merge_*^b^	0.084	0.112
Reflections used in refinement^a^	74,191 (6940)	55,413 (5244)
Reflections used for R-free^a^	3,738 (333)	2,775 (264)
*R_work_*^c^,^a^	0.1331 (0.2088)	0.1475 (0.2140)
*R_free_*^d^,^a^	0.1483 (0.2396)	0.1687 (0.2430)
Number of non-hydrogen atoms	2,026	2,146
Macromolecules	1,808	1,933
Ligands	19	11
Solvent	199	202
Protein residues	211	239
RMS (bonds) (Å)^e^	0.011	0.012
RMS (angles) (°)^e^	1.09	1.09
Ramachandran favored (%)	97.56	98.24
Ramachandran allowed (%)	2.44	1.76
Ramachandran outliers (%)	0.00	0.00
Rotamer outliers (%)	0.98	0.48
Clashscore	1.37	2.36
Average B-factor (Å^2^)	22.75	20.67
Macromolecules (Å^2^)	21.24	19.36
Ligands (Å^2^)	31.98	12.99
Solvent (Å^2^)	35.59	33.67

**Notes:**

a Parentheses indicate high resolution shell.

b Rmerge =∑_j_∑_h_| *I*_hj_ – < *I*_h_>| /∑_j_∑_h_ <*I*_h_>×100.

c R =∑||Fobs| – |Fcalc||/|Fobs|×100.

d work R_free_, based on 2%–5% of the total reflections.

e RMS deviation from ideality.
